# Collagen I Modifies Connexin-43 Hemichannel Activity via Integrin α2β1 Binding in TGFβ1-Evoked Renal Tubular Epithelial Cells

**DOI:** 10.3390/ijms22073644

**Published:** 2021-03-31

**Authors:** Joe A. Potter, Gareth W. Price, Chelsy L. Cliff, Colin R. Green, Paul E. Squires, Claire E. Hills

**Affiliations:** 1 Joseph Banks Laboratories, School of Life Sciences, University of Lincoln, Lincoln LN6 7DL, UK; jpotter@lincoln.ac.uk (J.A.P.); gprice@lincoln.ac.uk (G.W.P.); CCliff@lincoln.ac.uk (C.L.C.); PSquires@lincoln.ac.uk (P.E.S.); 2 Department of Ophthalmology and New Zealand National Eye Centre, University of Auckland, Auckland 1142, New Zealand; c.green@auckland.ac.nz

**Keywords:** collagen, extracellular matrix, connexin, hemichannel, ATP, Chronic Kidney Disease, inflammation, fibrosis, TGFβ1

## Abstract

Chronic Kidney Disease (CKD) is associated with sustained inflammation and progressive fibrosis, changes that have been linked to altered connexin hemichannel-mediated release of adenosine triphosphate (ATP). Kidney fibrosis develops in response to increased deposition of extracellular matrix (ECM), and up-regulation of collagen I is an early marker of renal disease. With ECM remodeling known to promote a loss of epithelial stability, in the current study we used a clonal human kidney (HK2) model of proximal tubular epithelial cells to determine if collagen I modulates changes in cell function, via connexin-43 (Cx43) hemichannel ATP release. HK2 cells were cultured on collagen I and treated with the beta 1 isoform of the pro-fibrotic cytokine transforming growth factor (TGFβ1) ± the Cx43 mimetic Peptide 5 and/or an anti-integrin α2β1 neutralizing antibody. Phase microscopy and immunocytochemistry observed changes in cell morphology and cytoskeletal reorganization, whilst immunoblotting and ELISA identified changes in protein expression and secretion. Carboxyfluorescein dye uptake and biosensing measured hemichannel activity and ATP release. A Cytoselect extracellular matrix adhesion assay assessed changes in cell-substrate interactions. Collagen I and TGFβ1 synergistically evoked increased hemichannel activity and ATP release. This was paralleled by changes to markers of tubular injury, partly mediated by integrin α2β1/integrin-like kinase signaling. The co-incubation of the hemichannel blocker Peptide 5, reduced collagen I/TGFβ1 induced alterations and inhibited a positive feedforward loop between Cx43/ATP release/collagen I. This study highlights a role for collagen I in regulating connexin-mediated hemichannel activity through integrin α2β1 signaling, ahead of establishing Peptide 5 as a potential intervention.

## 1. Introduction

In 2017, a report from the ‘Global Burden of Disease’ (GBD) study suggested that the incidence of Chronic Kidney Disease (CKD) was 9.1% (697.5 million cases) [1]. Worldwide, there are >2 million people on renal replacement therapy with the number of individuals on dialysis projected to increase to 5 million by 2030 [2]. CKD represents a global problem, with high-income countries typically spending more than 2–3% of their annual health-care budget on the treatment of end-stage kidney disease [2,3]. Loss of organ function in CKD is driven by sustained inflammation and progressive fibrosis [4,5]. However, our lack of understanding for how this pathology develops has hindered our ability to intervene clinically [6]. Consequently, new and more effective therapeutic approaches are urgently required.

In recent years, altered connexin (Cx) mediated communication has been linked to the inflammatory response in multiple tissue types [7,8,9,10]. Moreover, evidence suggests that these proteins represent therapeutic targets for treatment of conditions of chronic inflammation. Connexins are membrane bound proteins that facilitate cell-to-cell communication through their ability to oligomerise into hexameric connexons [11]. When neighbouring connexons align, they form gap junctions that provide a direct route for the transfer of small molecules and ions. In contrast to gap junctions, which typically open under physiological conditions, unbound connexons, or hemichannels, have a low open probability and open in response to injury. Hemichannels permit the release of small molecules (<1 kDa), e.g., adenosine triphosphate (ATP), into the local extracellular environment, where it can influence neighbouring cells via activation of purinoreceptors [12]. Expression of the purinergic P2X7 receptor (P2X7R) is upregulated in renal tubules of individuals with diabetic kidney disease [13], and has been heavily implicated in both progression of fibrosis in the kidney [14,15,16,17] and in other tissue types [18,19,20,21].

Renal fibrosis develops in response to increased deposition of the extracellular matrix (ECM), infiltration of immune cells and persistent activation of matrix producing fibroblasts [22]. A structural network of collagens, glycoproteins and proteoglycans, the ECM represents a dynamic environment that provides a structural scaffold and facilitates various cellular events including proliferation, differentiation, adhesion, migration and cell signalling [23]. Although devoid of intrinsic enzymatic activity, membrane proteins called integrins and integrin-mediated adhesions serve as bidirectional hubs, capable of transmitting environmental cues and/or signals between cells and their surroundings [24]. Consequently, cells adapt and respond to injury on the proviso that the dynamic state of the ECM is strictly maintained to meet the demands of the cell [25]. In the face of sustained injury, a loss of regulated ECM degradation/synthesis is paralleled by ECM remodelling and deposition [26]. This self-perpetuating state manifests as fibrosis and is considered a marker of disease progression, with remodelling of the ECM linked to the underlying pathology of various diseases, including cancer [27], osteoarthritis [28], liver cirrhosis [29] and chronic obstructive pulmonary disease [30].

Connexin mediated communication has been associated with fibrosis in various tissue types [13,31,32,33,34], with ECM remodelling recorded in multiple models of kidney injury. Several studies identify connexin-43 (Cx43) in the underlying pathology of both glomerular [35] and tubule disease [8], where severity of fibrosis in the kidney tubules dictates disease progression [36]. As the predominant isoform in the kidney, Cx43 expression is increased in renal biopsy material from individuals with both glomerular disease [35] and diabetic nephropathy [8], whilst heterozygous mice (*Cx43^+/−^*) induced to develop glomerulonephritis (GN) show signs of tubular dilation, decreased monocyte infiltration and reduced interstitial renal fibrosis [35]. Moreover, pharmacological intervention studies observed that a Cx43-specific antisense oligodeoxynucleotide improved structural and functional renal parameters in Cx43^+/+^ mice [37]. A similar protective effect was also observed in the heterogenous Cx43^+/−^ mouse induced with unilateral ureteral obstruction (UUO) [13,37] and in the RenTg model of hypertension induced CKD. When crossbred with Cx43^+/−^ mice, the RenTgCx43^+/−^ mouse model displayed a marked decrease in the mRNAs of Transforming Growth Factor β1 (TGFβ1) and collagen I as compared to RenTgCx43^+/+^ mice, whilst Sirius red staining confirmed diminished levels of interstitial collagen I accumulation [37]. Widely accepted as a major component of fibrotic tissues [37,38,39,40], upregulation of collagen I in the kidney is considered an early marker of renal fibrosis [41,42,43]. In wildtype UUO mice, Cx43 antisense attenuates ERK induced phosphorylation of SP1, a known activator of collagen I transcription. Paralleled by diminished mRNA levels of collagen I, collectively these observations suggest a strong link between aberrant Cx43 expression and collagen I deposition [37]. Our recent studies determined that TGFβ1-evoked changes in Cx43 expression are paralleled by increased hemichannel mediated ATP release [8]. Linked to inflammation and fibrosis in multiple tissue types [9,16,44,45], elevated ATP induced a number of changes indicative of tubular injury, that were in part blunted in both the Cx43^+/−^ UUO mouse and in vitro when primary proximal tubule cells were co-incubated with Cx43 hemichannel blocker Peptide 5 [13]. Despite our understanding that ECM remodeling impacts on cell phenotype [27,28,29,30], coupled with evidence that aberrant Cx43 communication is linked to increased collagen deposition [37], we are yet to determine if the ECM protein collagen I impacts on cell function via regulation of Cx-mediated hemichannel ATP release. Moreover, we do not know if/how collagen I and TGFβ1 together elicit a pathophysiological effect. Consequently, in the present study we determine if positive feedback exists between Cx43 hemichannel mediated ATP release and collagen I secretion/deposition. Using a series of complementary strategies to assess protein expression and function, we demonstrate that human kidney (HK2) proximal tubule epithelial cells exhibit increased hemichannel opening and a rise in ATP release when cultured on collagen I. Interestingly, these effects are exacerbated when cells are co-incubated with TGFβ1 and blunted when pre-incubated with either a neutralising antibody to integrin α2β1 or Cx43 hemichannel blocker Peptide 5. We then assess this relationship and its implications on cell function, by screening for expression changes in markers of tubular injury in cells cultured on uncoated versus collagen coated plastic. Importantly, collagen I/TGFβ1 induced changes to markers of tubular injury, e.g., Integrin Linked Kinase-1 (ILK1) and N-cadherin are partly ablated, when either cell-collagen attachment or Cx43 hemichannel activity is blocked. Furthermore, linked to increased collagen I deposition in the Cx43^+/−^ mouse [37], TGFβ1 induced collagen I secretion was blunted by Peptide 5. Collectively these data support a role for Cx43 hemichannel mediated ATP release in driving changes of tubular injury. Importantly data using the hemichannel blocker Peptide 5, suggests that targeting this aberrant hemichannel activity may be of future therapeutic interest.

## 2. Results

### 2.1. TGFβ1 Increases Expression of ECM Proteins and Alters Cell–Substrate Interactions

**Figure 1:**
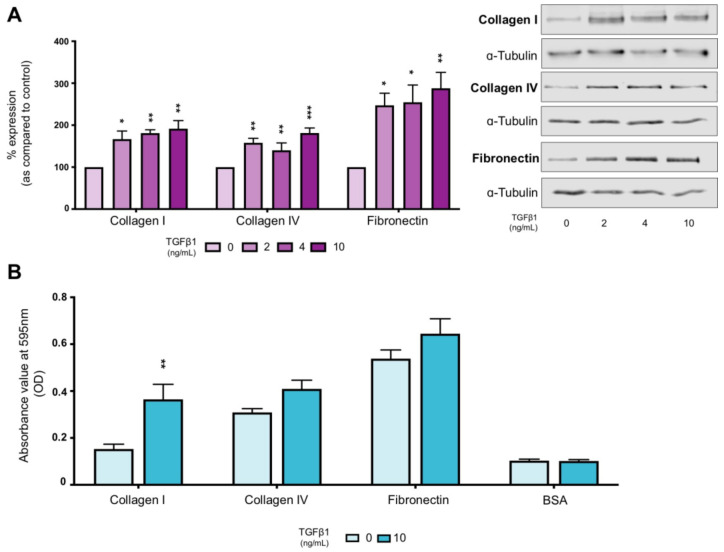
TGFβ1 increases ECM expression and affinity. Whole cell expression of collagen I, collagen IV and fibronectin (**A**) were confirmed via immunoblotting Representative blots for each protein are shown, with expression normalised against α-tubulin as a loading control. Data are presented as mean ± SEM, *n* = 5 with key significances shown (* *p* < 0.05, ** *p* < 0.01, *** *p* < 0.001 vs. control). Cell affinity for binding to collagen I, collagen IV and fibronectin were analysed by a cell adhesion assay (**B**). BSA was used as a negative control. Data are presented as mean ± SEM, *n* = 4 with key significances shown: (** *p* < 0.01 vs. control).

Cells were stimulated with TGFβ1 (2–10 ng/mL) for 48 h and whole cell expression of ECM proteins were assessed (Figure 1A). TGFβ1 increased expression of collagen I to 166.6 ± 19.4%, 181.2 ± 7.9% and 191.7 ± 19.2% and collagen IV to 157.1 ± 10.9%, 160.0 ± 13.7% and 189.8 ± 10.8% at 2, 4 and 10 ng/mL, respectively, as compared to control. Expression of fibronectin increased to 266.9 ± 27.5%, 274.8 ± 46.4% and 290.8 ± 38.1% at 2, 4 and 10 ng/mL TGFβ1, respectively, as compared to control (Figure 1A). Using an ECM cell affinity assay (Figure 1B) we determined that cells significantly increase affinity for collagen I in the presence of TGFβ1 (0.36 ± 0.06 OD) as compared to cells cultured on collagen I alone (0.15 ± 0.02 OD). This effect is most likely mediated by increased cell surface expression of integrin α2 in TGFβ1 treated cells (see Figure S1), one of two key α subunits for which collagen I favours binding. When cultured on collagen IV or fibronectin, co-incubation with TGFβ1 failed to significantly alter the binding affinity between cells and their substrate (Figure 1B).

### 2.2. Collagen I and TGFβ1 Stimulate Aberrant Cell Morphology and Cytoskeletal Reorganization

**Figure 2:**
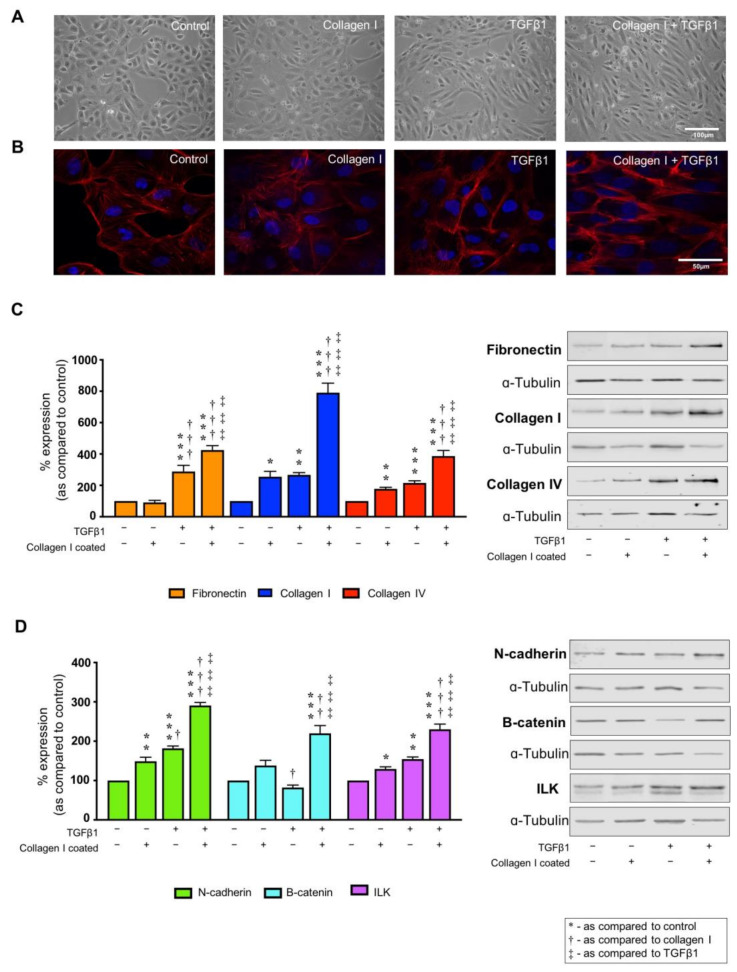
Collagen I exacerbates TGFβ1-evoked changes in expression of markers of tubular injury. Phase contrast microscopy (**A**) and TRITC-conjugated phalloidin (**B**) assessed changes in cell morphology (magnification × 20) and cytoskeletal reorganisation (magnification × 63), respectively, when cultured on collagen I (50 µg/mL) with or without TGFβ1 (10 ng/mL). Whole cell expression of fibronectin, collagen I and collagen IV (**C**) and N-cadherin, β-catenin and ILK (**D**) were confirmed via immunoblotting. Uncoated plastic served as a control. Representative blots for each protein are shown, with expression normalised against α-tubulin as a loading control. Data are presented as mean ± SEM, *n* = 5 with key significances shown (* *p* < 0.05, ** *p* < 0.01, *** *p* < 0.001; ^†^ *p* < 0.05, ^††^ *p* < 0.01, ^†††^ *p* < 0.001 vs. collagen I coated; ^‡^ *p* < 0.05, ^‡‡‡^ *p* < 0.001 vs. TGFβ1).

TGFβ1 (10 ng/mL) altered the morphology from a classic epithelial cobblestone appearance towards a more elongated cell architecture consistent with fibroblasts. The effects were exacerbated by culturing the cells on collagen I (Figure 2A). This shift in gross morphology was paralleled by cytoskeletal reorganisation, with F-actin stress fibres appearing at the cell periphery in cells cultured on collagen I and stimulated with TGFβ1 (Figure 2B). In cells cultured on collagen I alone only partial stress fibre formation was observed.

### 2.3. TGFβ1 Alters Markers of Tubular Injury, an Effect Exacerbated by Co-Culture on Collagen I

ECM remodelling and altered cell–substrate interactions are crucial not only for cell anchorage, but also in regulating several cell functions [46,47,48,49]. To determine if collagen I impacts on cell behaviour, immunoblotting determined whole cell expression of proteins central to tubulointerstitial fibrosis in lysates from cells cultured on collagen I alone or following co-incubation with TGFβ1 (10 ng/mL) for 48 h. As expected, the pro-fibrotic cytokine increased expression of fibronectin to 314.8 ± 41.4% as compared to control, whilst minimal changes were observed in cells cultured on collagen I alone (Figure 2C). Interestingly, when cells cultured on collagen I were stimulated with TGFβ1, fibronectin expression was further enhanced to 425 ± 28.5% compared to uncoated control. Expression of collagen I (255 ± 34.2%) and collagen IV (177.7 ± 9.9%,) were significantly increased in cells cultured on the ECM substrate, an effect markedly exacerbated by co-incubation of cells with TGFβ1 to 790.7 ± 60.8% (collagen I) and 386.9 ± 35.4% (collagen IV).

When cultured on collagen I, expression of adherens junction protein N-cadherin increased to 149 ± 10.2%. The effect was significantly amplified by co-incubation with TGFβ1 (290.8 ± 7.7%) as compared to TGFβ1 stimulated cells on uncoated control (181.7 ± 6.2%) (Figure 2D). The adherens junction protein and mediator of Wnt signalling β-catenin, exhibited increased expression of 220.0 ± 19.8% in TGFβ1-treated cells cultured on collagen I as compared to uncoated control (Figure 2D). Moreover, expression of ILK, an upstream regulator of β-catenin, increased in cells cultured on collagen I to 129.4 ± 5.6% and in TGFβ1 treated cells (154.4 ± 5.5%), whilst co-incubation of the pro-fibrotic cytokine and collagen I evoked the largest change compared to uncoated unstimulated control (230.2 ± 13.4%; Figure 2D). As a control experiment, cells cultured on fibronectin or collagen IV in the presence/absence of TGFβ1 have little effect on expression changes in collagen IV, collagen I or β-catenin (see Figure S2).

### 2.4. TGFβ1 Exacerbates Collagen I Induced Hemichannel Activity and Cx43 Mediated ATP Release

Recent studies suggest that ECM remodelling has been linked to changes in cell behaviour and function through altered connexin-mediated cell communication [50,51,52,53,54]. On this basis, a role for collagen I in mediating tubular injury through regulating connexin-mediated hemichannel activity was assessed.

**Figure 3:**
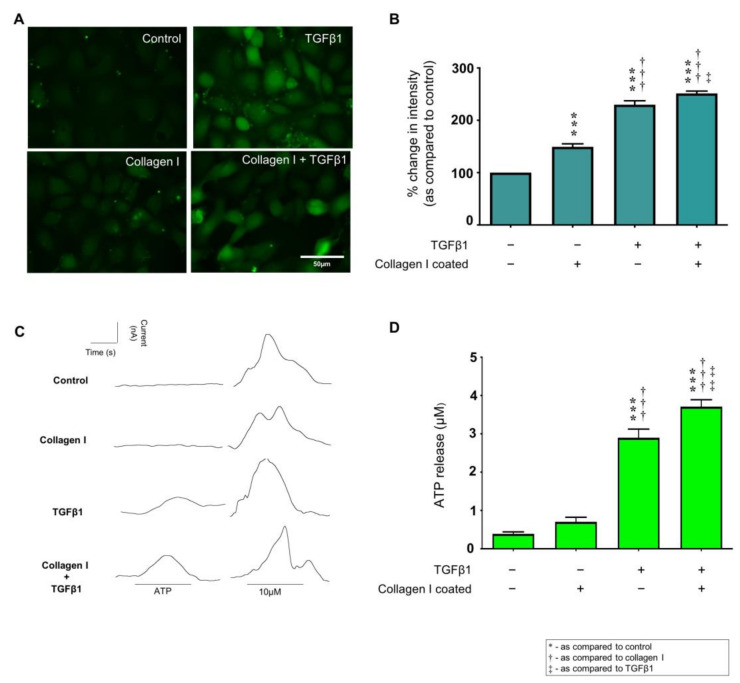
Collagen I and TGFβ1 co–stimulate hemichannel mediated ATP release. A carboxyfluorescein dye uptake assay (**A**,**B**) assessed changes in hemichannel activity. Biosensors were used to measured hemichannel mediated ATP release (**C**,**D**). Representative traces are shown (**C**). ATP peaks were measured and compared to uncoated control that exhibited a negligible response following removal of extracellular calcium. Exogenous ATP (10 mM) was used for calibration. Bio–sensing data are expressed as mean ± SEM *n* = 5 of multiple cells (**D**). Data are presented as mean ± SEM, *n* = 5 with key significances shown (*** *p* < 0.001 vs. control; ^†††^ *p* < 0.001 vs. collagen I coated; ^‡^ *p* < 0.05, ^‡‡^ *p* < 0.01 vs. TGFβ1). Uncoated plastic served as a control.

Increased carboxyfluorescein dye uptake reflects increased expression of functional hemichannels and was observed in cells cultured on collagen I (149.3 ± 5.9%). TGFβ1 increased dye uptake to 229.9 ± 7.4% as compared to control. The combination of collagen I and TGFβ1 amplified the effect (251.4 ± 4.5%; Figure 3A,B). To match changes in expression to function, biosensing was used to measure real time changes in extracellular ATP. Cultured as above, the concentration of extracellular ATP increased to 2.9 ± 0.22 µM in TGFβ1 treated cells and to 3.7 ± 0.18 µM in TGFβ1 treated cells cultured on collagen I, as compared to uncoated control (0.4 ± 0.05 µM; Figure 3C,D). There was a 79% increase in ATP release from cells cultured on collagen I alone (0.7 ± 0.12 µM), but this was not statistically significant compared to uncoated control.

### 2.5. Collagen I ± TGFβ1-Evoked Hemichannel Activity Is Partially Mediated via Integrin α2β1 Binding

**Figure 4:**
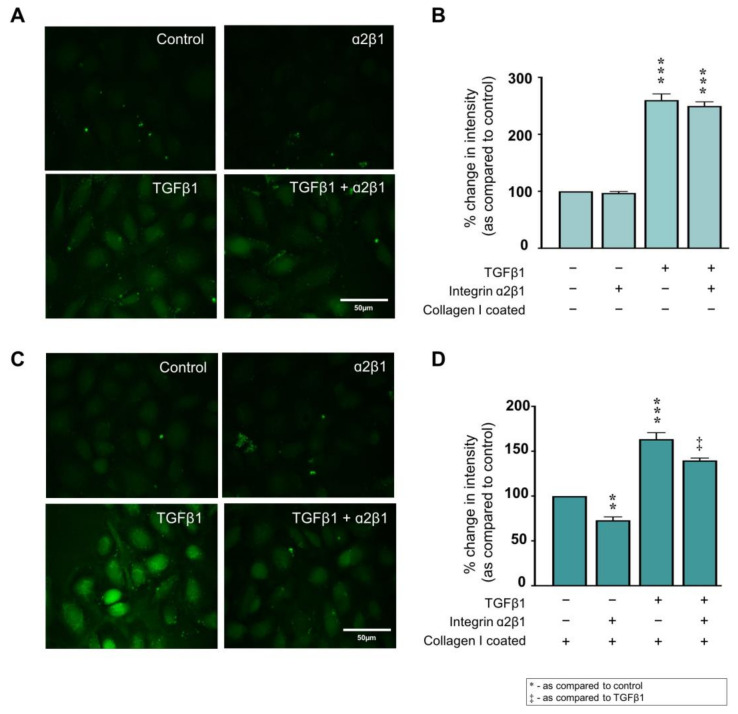
Integrin α2β1 binding partially mediates collagen I ± TGFβ1-evoked hemichannel activity. A carboxyfluorescein dye uptake assay assessed changes in hemichannel activity in cells cultured on uncoated (**A**) and collagen coated (**C**) plastic ± TGFβ1 (10 ng/mL) ± anti–integrin α2β1 neutralising antibody (2.5 µg/mL). Pixel intensity of dye loading was quantified and compared to control for ~15 cells in four separate experiments (**B**,**D**). Data is presented as mean ± SEM, *n* = 4 with key significances shown (** *p* < 0.01, *** *p* < 0.001 vs. control; ^‡^ *p* < 0.05 vs. TGFβ1).

Increased collagen I deposition and altered activity of the principal binding integrin α2β1 have been associated with the onset of fibrosis in several disease states [55,56,57,58], including CKD [59]. To determine if collagen I modulates connexin-mediated hemichannel activity through integrin α2β1 binding, HK2 cells were cultured on uncoated or collagen I coated plastic in low (5 mmol/L) glucose for 48 h prior to overnight serum starvation. Cells were stimulated with TGFβ1 (10 ng/mL) ± anti-integrin α2β1 neutralising antibody (2.5 µg/mL) for 48 h and dye uptake assessed. Co-incubation with an anti-integrin α2β1 neutralising antibody had minimal effect (249.7 ± 7.2%) on dye uptake in TGFβ1 treated cells (260.1 ± 10.9%) (Figure 4A,B), whilst blocking α2β1 significantly reduced dye uptake to 73.0 ± 3.8% in cells cultured on collagen I compared to collagen alone. TGFβ1 treated cells cultured on collagen I and co-incubated with the neutralising antibody demonstrated a partial reduction in dye uptake from 163.5 ± 7.2% to 139.7 ± 2.6% as compared to uncoated control cells (Figure 4C,D).

### 2.6. Collagen I Partly Regulates Markers of Tubular Injury through Integrin α2β1-Mediated Signal Transduction

**Figure 5:**
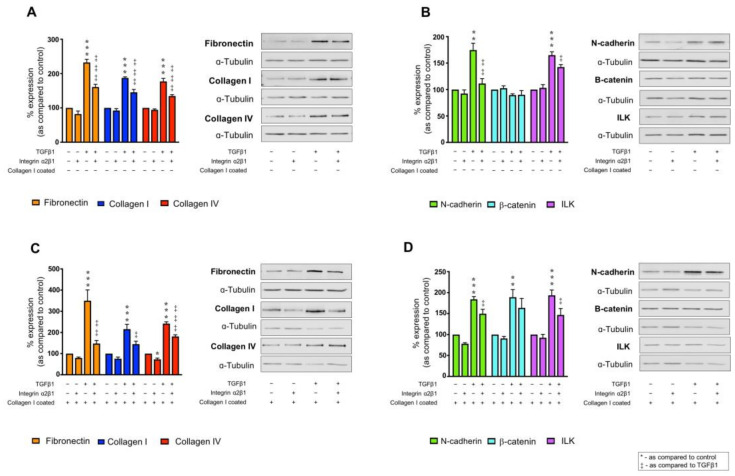
Inhibition of integrin α2β1 regulates collagen I ± TGFβ1-evoked changes in markers of tubular injury. HK2 cells were cultured on uncoated (**A**,**C**) or collagen I (50 µg/mL) coated (**C**,**D**) plastic in low glucose (5 mmol/L) ± TGFβ1 (10 ng/mL) ± anti-integrin α2β1 neutralising antibody (2.5 µg/mL) for 48 h. Whole cell expression of fibronectin, collagen I and collagen IV (**A**,**C**) and N­–cadherin, β–catenin and ILK (**B**,**D**) were confirmed via immunoblotting. Representative blots for each protein are shown, with expression normalised against α–tubulin as a loading control. Data are presented as mean ± SEM, *n* = 4 with key significances shown (* *p* < 0.05, ** *p* < 0.01, *** *p* < 0.001 vs. control; ^‡^ *p* < 0.05, ^‡‡^ *p* < 0.01, ^‡‡‡^ *p* < 0.001 vs. TGFβ1).

When cultured on uncoated plastic, co-incubation with an anti-integrin α2β1 neutralising antibody decreased expression of fibronectin (232.3 ± 9.5% to 160.8 ± 7.8%), collagen I (232.3 ± 9.5% to 160.8 ± 7.8%) and collagen IV (177.2 ± 8.9% to 134.6 ± 4.0%) as compared to control in TGFβ1 treated cells (Figure 5A). Moreover, blocking integrin α2β1 activity blunted the TGFβ1-evoked increase in N-cadherin expression (174.7 ± 13.0% to 111.5 ± 9.1%) and ILK (165.0 ± 6.3% to 142.5 ± 4.6%) as compared to control (Figure 5B). No change in β-catenin expression was observed.

When cultured on collagen I and stimulated with TGFβ1, fibronectin and collagen I expression increased to 349.9 ± 51.0% and 215.6 ± 22.9%, respectively. This increased expression was significantly reduced to 147.2 ± 15.0% (fibronectin) and 144.5 ± 13.9% (collagen I) when a2β1 activity was blocked (Figure 5C). Interestingly, inhibition of integrin α2β1 in the absence of TGFβ1 decreased collagen IV expression to 72.7 ± 6.2%, whilst co-incubation of TGFβ1 and α2β1 neutralising antibody negated the TGFβ1-evoked increases in collagen IV from 241.8 ± 8.2% to 180.9 ± 7.8% compared to control (Figure 5C). Blocking integrin α2β1 reversed the TGFβ1-evoked increase in injury markers N-cadherin (184.0 ± 6.6% to 149.7 ± 10.5%) and ILK (193.7 ± 13.0% to 146.8 ± 15.3%) as compared to control cells cultured on collagen I alone (Figure 5D). However, there was no effect on collagen I + TGFβ1-evoked increases in β-catenin expression (189.1 ± 18.2%; Figure 5D).

### 2.7. Peptide 5 Negates Collagen I ± TGFβ1-Induced Cx43 Hemichannel Activity

**Figure 6:**
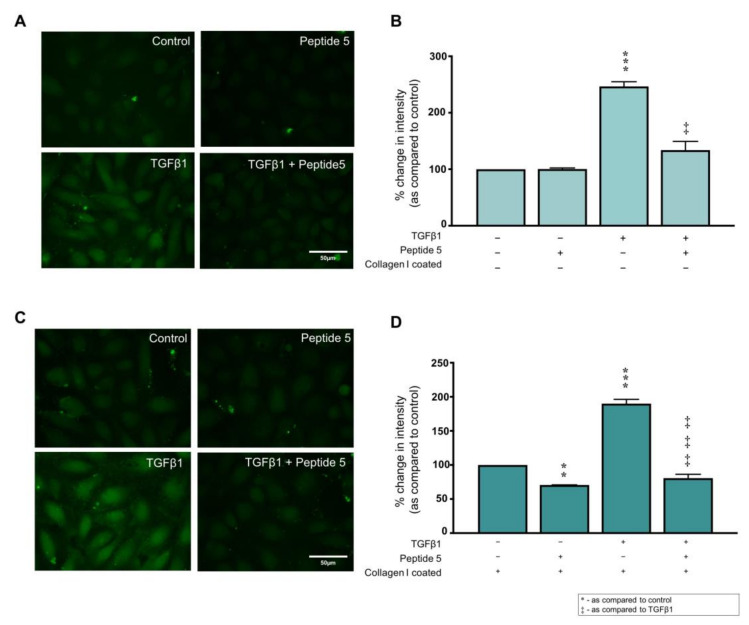
Incubation with Peptide 5 inhibits collagen I ± TGFβ1-induced changes in hemichannel activity. HK2 cells were cultured on uncoated or collagen I (50 µg/mL) coated fluorodishes in low glucose (5 mmol/L) ± TGFβ1 (10 ng/mL) ± Peptide 5 (25 µM) for 48 h. A carboxyfluorescein dye uptake assay assessed changes in hemichannel activity on both uncoated plastic (**A**) and collagen coated plastic (**C**) Pixel intensity of dye loading was quantified (**B**,**D**) and compared to control for 15 cells in four separate experiments Data are presented as mean ± SEM, *n* = 4 with key significances shown (** *p* < 0.01, *** *p* < 0.001 vs. control; ^‡^ *p* < 0.05, ^‡‡‡^ *p* < 0.001 vs. TGFβ1).

Peptide 5 is a Cx43 mimetic with beneficial effects in multiple models of injury [60,61,62,63,64,65,66]. We employed Peptide 5 to determine if collagen I mediates its effects on expression of proteins associated with tubular injury via Cx43 hemichannel activity. Peptide 5 (25 µM) blocked the TGFβ1 (10 ng/mL)-induced increase in dye uptake from 246.6 ± 8.4% to 133.9 ± 15.4%, as compared to uncoated control (Figure 6A,B). In cells cultured on collagen I, Peptide 5 decreased hemichannel activity to 70.7 ± 0.2%, an effect recapitulated when Peptide 5 was co-incubated with TGFβ1 stimulated cells, where dye uptake decreased from 190.0 ± 6.3% to 80.7 ± 5.7 as compared to collagen I coated control (Figure 6C,D). Scrambled Peptide 5 has no effect on TGFβ1 induced dye uptake [13]

### 2.8. Peptide 5 Negates TGFβ1 ± Collagen I-Induced Tubular Injury

**Figure 7:**
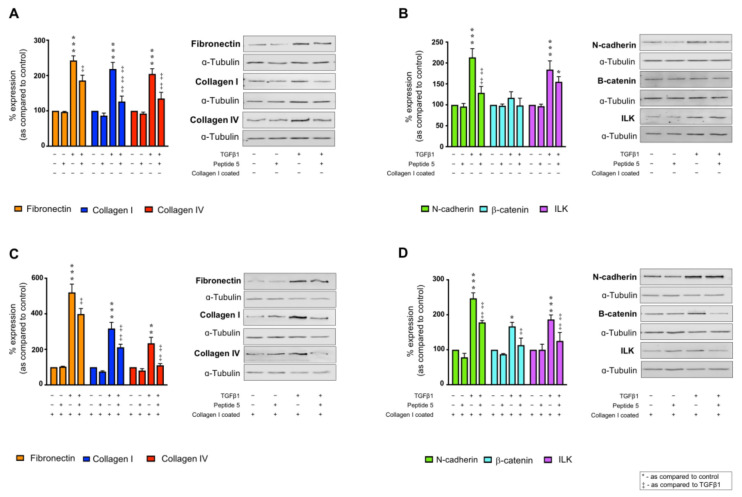
Peptide 5 negates TGFβ1 ± collagen I increased expression of markers of tubular injury. HK2 cells were cultured on uncoated (**A**,**B**) or collagen I (50 µg/mL) coated (**C**,**D**) plastic in low glucose (5 mmol/L) ± TGFβ1 (10 ng/mL) ± Peptide 5 (25 µM) for 48 h. Whole cell expression of fibronectin, collagen I and collagen IV (**A**,**C**) and N–cadherin, β–catenin and ILK (**B**,**D**) were confirmed via immunoblotting. Representative blots for each protein are shown, with expression normalised against α-tubulin as a loading control. Data are presented as mean ± SEM, *n* = 5 with key significances shown (* *p* < 0.05, ** *p* < 0.01, *** *p* < 0.001 vs. control; ^‡^ *p* < 0.05, ^‡‡^ *p* < 0.01, ^‡‡‡^ *p* < 0.001 vs. TGFβ1).

Having established that Peptide 5 prevents collagen I &/or TGFβ1 induced Cx43 mediated hemichannel activity, the impact of blunting Cx43 cell–cell communication on collagen I ± TGFβ1 induced expression changes were further assessed. Co-incubation of TGFβ1 and Peptide 5 reduced expression of fibronectin from 242.5 ± 13.2% to 185.5 ± 15.0% and collagen I from 218.3 ± 18.8% to 126.3 ± 15.5% in cells cultured on uncoated plastic as compared to control (Figure 7A). Similarly, TGFβ1 treated cells demonstrated an increase in collagen IV expression to 204.4 ± 14.8%, an effect blunted by Peptide 5 to 134.9 ± 17.5% (Figure 7A). Peptide 5 incubation did not alter protein expression in the absence of TGFβ1 but did abrogate the TGFβ1-evoked increase in N-cadherin and ILK from 213.4 ± 20.8% and 184.2 ± 20.9% to 128.7 ± 15.3% and 155.1 ± 12.4%, respectively, as compared to uncoated control (Figure 7B). No change in β-catenin expression was observed.

When cultured on collagen and stimulated with TGFβ1, Peptide 5 blunted the previously observed synergistic response with fibronectin expression reduced from 520.4 ± 46.7% to 399.0 ± 31.1%, collagen I from 317.4 ± 34.6% to 211.7 ± 17.2% and collagen IV 234.3 ± 33.7% to 110.3 ± 9.4% as compared to collagen I cultured cells (Figure 7C). Peptide 5 also reduced TGFβ1-stimulated increases in N-cadherin (247.0 ± 15.8%), β-catenin (166.9 ± 11.7%) and ILK (186.6 ± 12.9%) to 178.3 ± 5.4%, 107.7 ± 10.9% and 113.3 ± 20.0%, respectively, as compared to cells cultured on collagen I alone (Figure 7D).

### 2.9. Peptide 5 Reduces TGFβ1-Induced Collagen I Secretion

**Figure 8:**
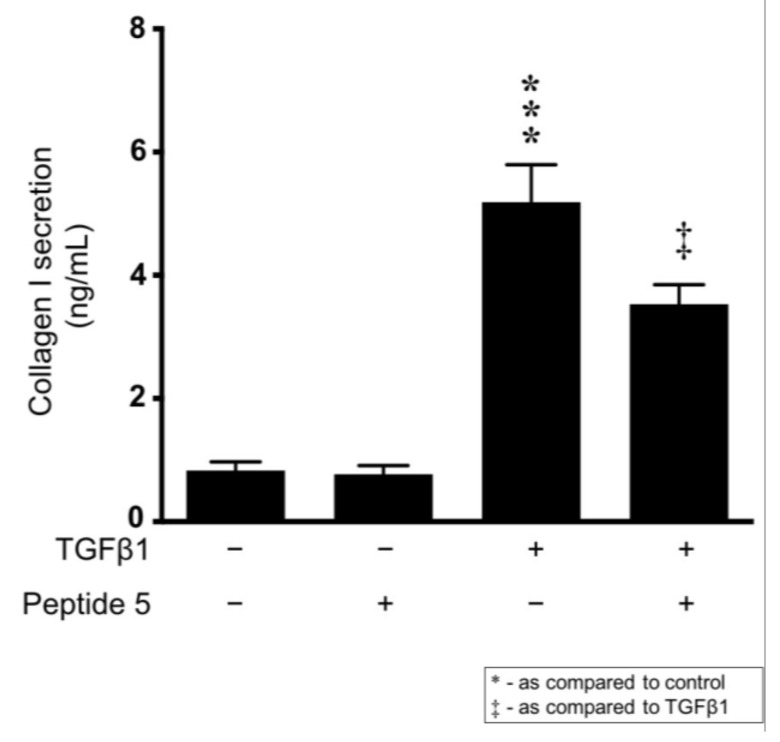
Peptide 5 negates TGFβ1–induced changes in collagen I secretion. HK2 cells were cultured in low glucose (5 mmol/L) ± TGFβ1 (10 ng/mL) ± Peptide 5 (25 µM) for 48 h. Collagen I secretion was quantified via an ELISA using treated HK2 cell supernatant. Data are presented as mean ± SEM, *n* = 4 with key significances shown (*** *p* < 0.001 vs. control; ^‡^ *p* < 0.05 vs. TGFβ1).

With evidence demonstrating that inhibiting integrin a2β1 reduces TGFβ1-induced changes in both hemichannel activity and protein expression on uncoated control, our data suggest that TGFβ1 increases collagen I secretion. Moreover, with evidence that Cx43 activity can enhance collagen I expression through attenuation of ERK induced SP1 activation [37], we assessed a role for Cx43 hemichannel activity in mediating TGFβ1 induced collagen I secretion. TGFβ1 (10 ng/mL) significantly increased collagen I secretion from 0.83 ± 0.14 ng/mL to 5.19 ± 0.61 ng/mL. Co-incubation of Peptide 5 (25 µM) partially reversed this TGFβ1-evoked increase to 3.53 ± 0.31 ng/mL (Figure 8).

## 3. Discussion

Chronic Kidney Disease is characterised by multiple structural and functional disturbances culminating in loss of renal function and ultimately organ failure [67]. Irrespective of aetiology, tubulointerstitial fibrosis represents a final common pathway, with interstitial deposition of extracellular matrix dictating severity of injury and disease progression [68]. Despite this, current treatment focuses on lifestyle intervention, regulation of blood glucose levels and normalisation of blood pressure [3]. Consequently, management options for late-stage damage are limited to both dialysis and transplantation. In the absence of a definitive treatment, understanding mechanisms that promote fibrosis in and around the kidney tubules are essential in the search for future therapeutic targets.

Recently, a group of transmembrane proteins called connexins have attracted considerable interest as a potential target for treatment of multiple disease states [61,69,70,71,72,73], including CKD [13,34,74]. In the kidney, fibrosis develops in response to an imbalance between excessive extracellular matrix synthesis and degradation. Consequently, the basement membrane on which tubule epithelial cells sit is damaged and cells become exposed to extracellular matrix proteins and activated cytokines (e.g., TGFβ1) [5]. Together, these matrix proteins and cytokines induce membrane receptor-ECM attachment, altered signal transduction and ultimately a switch in cell phenotype [26]. Consequently, cross talk between profibrotic TGFβ, integrins and the ECM is pivotal to the development and progression of renal fibrosis [59,75,76,77]. Work using different models of kidney disease have identified a strong correlation between the primary tubular connexin isoform (Cx43) and the ECM protein, collagen I [35,37]. However, it is unknown if collagen I and Cx43 exhibit a reciprocal relationship. To better understand if the ECM protein drives a loss of epithelial stability through Cx43 mediated cell–cell communication, we adopted an in vitro approach in which human proximal tubule epithelial cells were cultured on uncoated versus collagen I coated plastic ± TGFβ1. We further employed the Cx43 mimetic, Peptide 5, to delineate a role for Cx43 hemichannel mediated ATP release in driving these effects. Initial studies demonstrated that TGFβ1 evoked a concentration-dependent increase in whole cell expression of the ECM proteins fibronectin, collagen I and collagen IV, and that binding affinity to each of these proteins is increased in TGFβ1-treated cells. Interestingly, cells bound most readily to collagen I, an attachment mediated by integrin a2β1 [78]. Known to positively regulate collagen expression, genetic deletion of the integrin α2 subunit or selective antagonism of the integrin protects mice from the development of glomerular fibrosis after partial renal ablation or adriamycin administration [55]. Furthermore, in the UUO-induced model of advanced interstitial inflammation and fibrosis, upregulation of integrin β1 expression precedes initiation of epithelial-to-mesenchymal transition (EMT), with blockade of integrin β1 activity protecting from renal fibrosis [79]. Integrin linked kinase (ILK1) is a key intracellular component of the integrin signaling complex and a downstream protein kinase of the integrin β1 subunit [80]. Furthermore, ILK1 has been demonstrated to play an active role in mediating tubular EMT and interstitial fibrosis [81,82]. Consequently, initial studies assessed expression of six markers of tubular injury, including ILK1 in cells cultured on collagen I ± TGFβ1 in the presence or absence of a neutralising antibody to the a2β1 integrin isoform. With the exception of β-catenin, TGFβ1 increased expression of all markers, an effect that was amplified when cells were cultured on collagen I. Moreover, inhibition of integrin a2β1 activity significantly blunted the collagen I ± TGFβ1 induced response, including that of the key signal transducer, ILK1. Whilst these data support the notion that collagen-induced a2β1/ILK signaling most likely participates in the progression of tubulointerstitial fibrosis, to evaluate a downstream role for connexin hemichannels in this response, carboxyfluorescein dye uptake and ATP biosensing were used to assess hemichannel activity and ATP release. When cells were cultured on collagen I or stimulated with TGFβ1 an increase in dye uptake was observed. This increase in hemichannel activity was paralleled by ATP release in TGFβ1 treated cells, an effect that was accentuated when the cells were cultured on collagen I. Interestingly, the increase in hemichannel activity was partly blunted when cells were pre-incubated with a neutralising antibody to the a2β1 integrin, the latter of which had no direct effect on cell morphology (see Figure S3). Furthermore, pre-incubation with Peptide 5 confirmed that hemichannel dye uptake was Cx43 specific.

Peptide 5 is a 12 amino acid peptide which targets the 2nd extracellular loop of Cx43 [60]. Peptide 5 has been shown to negate inflammation in various models of injury, where the underlying pathology is mediated by the Nod-Like Receptor Protein-3 (NLRP3) inflammasome [73,83]. Our earlier studies using a Cx43^+/-^ UUO model demonstrate that when Cx43 expression is reduced, tight junction (TJ) and adherens junction (AJ) protein expression is restored [13]. Disassembly of the TJ and AJ complex is thought to be an initiating factor in tubular injury, with loss of epithelial characteristics paralleled to increased ECM remodelling and upregulation of markers commonly associated with a mesenchymal phenotype [13,17,84]. A role for Cx43 hemichannel activity in mediating these effects was supported when Peptide 5 successfully prevented TGFβ1-evoked changes in the expression of junction proteins E-cadherin, N-cadherin, Claudin-2 and Zona-occludens-1 (ZO-1) in human renal proximal tubule cells [13].

In the current study, we demonstrate that pre-incubation with the Cx43 mimetic significantly negates collagen I ± TGFβ1 induced hemichannel activity, an effect paralleled by reduced expression of key proteins linked to fibrosis and tubular injury. Interestingly, TGFβ1 treated cells cultured on uncoated plastic exhibit rescued hemichannel activity and decreased expression of tubular injury markers when a2β1 activity was blocked.

Combined with earlier evidence from the Cx43^+/−^ mouse induced with UUO that demonstrates decreased collagen deposition [37], these data suggest that TGFβ1 not only increases whole cell expression of collagen I but also increases secretion into the ECM via a Cx43 dependent mechanism. To evaluate a role for Cx43 hemichannel mediated ATP release in collagen secretion, we performed a collagen I ELISA on TGFβ1 treated cells and observed a Peptide 5-sensitive increase in secretion. With decreased collagen deposition in the Cx43^+/−^ mouse paralleled by protection against EMT, it is plausible that when present with active TGFβ1, collagen I could cooperatively increase tubular epithelial cell damage via a2β1/ILK regulated Cx43 hemichannel activity. Lastly, whilst these experiments determine a synergistic role for collagen I and TGFβ1 in regulating Cx43 hemichannel activity, we concede that this in vitro data provides a minimalistic model of the multifactorial events that give rise to tubulointerstitial fibrosis and recommend caution in translating these novel findings to the in vivo situation. Whilst possible that Peptide 5 may improve kidney function by blocking Cx43 induced collagen secretion, ultimately negating a cycle of events which could perpetuate tubular damage, a better understanding of how the ECM remodelling may impact on cell function via regulation of hemichannel activity in vivo is now required.

## 4. Materials and Methods

### 4.1. Materials

Human Kidney (HK2) proximal tubule epithelial cells were derived from American Type Culture Collection (ATCC) (LGC Standards). Tissue culture plastic and supplies were attained from Corning Inc (New York, NY, USA) and Invitrogen (Paisley, UK). For Western blotting, Immobilon-FL was purchased from Millipore (Watford, UK), blocking buffer and fluorescent anti-rabbit and anti-mouse secondary antibodies were obtained from Licor Biosciences (Lincoln, NE, USA). Antibodies anti-collagen I (ab34710), anti-collagen IV (ab6586), anti-integrin α2β1 (ab24697), anti-connexin-43 (ab11370), and anti-N-cadherin (ab18203) were sourced from Abcam (Cambridge, UK). Anti-fibronectin (sc-9068) was purchased from Santa Cruz (California, USA) and anti-β-catenin (9582P) and anti-ILK (3862S) were from Cell Signalling (Danvers, Massachusetts, USA). The anti-procollagen I α1 ELISA (ab210966) was purchased from Abcam (Cambridge, UK), and the cytoselect cell adhesion assay (CBA-070) was from Cell Biolabs Inc (California, USA). Recombinant human transforming growth factor-β1, rat tail collagen type I solution, along with all immunocytochemistry and other general reagents were purchased from Sigma-Aldrich (Poole, UK). ATP biosensors were from Sarissa Biomedical Ltd. (Coventry, UK) and fluorodishes were from World Precision Instruments (WPI) (Hertfordshire, UK). Peptide 5 was purchased from Syn Peptide (Shanghai, China).

### 4.2. Cell Culture and Treatment

HK2 cells are a clonal proximal tubular epithelial cell line derived from normal kidney and immortalised by the transduction of human papilloma virus-16 (HPV-16) E6/E7 genes. Cells (passages 18–30) were maintained in Dulbecco’s Modified Eagle’s Medium DMEM/Ham’s F12 (DMEM/F12) medium, supplemented with Fetal Calf Serum (FCS) (10% wt/vol), penicillin-streptomycin (2%), epidermal growth factor (EGF) (5 ng/mL) and glutamine (2 mmol/L), and cultured at 37 °C in a humidified environment with 5% CO_2_. For all treatments, cells were cultured in low glucose DMEM/F12 (5 mmol/L) for 48 h, followed by overnight serum-starvation prior to treatment with TGFβ1 (2–10 ng/mL) ± anti-integrin α2β1 neutralising antibody (2.5 µg/mL), or Peptide 5 (25 µM) for 48 h. For collagen I coated plates, sterile rat tail collagen I (50 µg/mL) was added to the culture plate for 5 h and incubated in a humidified environment (37 °C, 5% CO_2_). Plates were left to air dry overnight and cells were seeded.

### 4.3. Immunocytochemistry

HK2 cells were stimulated with TGFβ1 (10 ng/mL) for 48 h. At 80% confluence, cells were fixed with paraformaldehyde (4%) and blocked for 1 h at room temperature (RT) with goat serum (10%) in PBS-Triton X-100 (0.01%). The nuclear stain 4′,6-diamidino-2-phenylindole (DAPI) (1 mmol/L) was added to each coverslip for 3 min. After 3 × 10-min washes with PBS-Triton X-100 (0.01%), cells were incubated for 1 h at RT with tetramethyl rhodamine isothiocyanate (TRITC)-conjugated phalloidin (1:400). The coverslips were mounted on glass slides using anti-fade Citifluor (glycerol/PBS). Immunofluorescence was visualised using a Leica TC SP8 confocal microscope (Wetzlar, Germany).

### 4.4. Western Blotting

Whole cell lysates were prepared and separated via SDS-PAGE gel electrophoresis and transferred onto Immobilon-FL PVDF membranes as previously described [85]. Membranes were blocked for 1 h at RT with Intercept blocking buffer (Licor), then probed overnight at 4 °C with specific polyclonal antibodies against collagen I (1:1000), collagen IV (1:1000), fibronectin (1:2000), N-cadherin (1:1000), β-catenin (1:2000), ILK (1:1000) or α-Tubulin (1:20,000) which was used as a housekeeping protein. After 4 × 5-min washes with PBS-Tween (0.01%), membranes were probed with corresponding anti-rabbit or anti-mouse secondary antibodies (1:20,000) at RT for 1 h. Bands were visualised using an Odyssey FC imaging unit and semi-quantified using ImageStudio software (v5.2, Licor).

### 4.5. Cell Adhesion Array

An ECM cell adhesion array (CBA-070) was performed according to manufacturer’s instructions. Briefly, HK2 cells were incubated with TGFβ1 (10 ng/mL) for 48 h. Cells were seeded at 0.5 × 10^6^ onto the 48-well plate and was left to incubate for 90 min at 37 °C, 5% CO_2_. The media was discarded, and the plate was washed 5 times with PBS. Cell stain solution was added for 10 min at RT and any residual stain was removed with 5 washes using dH_2_O and the plate was left to air dry. Using an orbital shaker, the plate was incubated for a final 10 min at RT with extraction solution. The optical density (OD) was read at 595 nm. Bovine serum albumin (BSA) was used as a negative control.

### 4.6. Collagen I Enzyme-Linked Immunosorbent Assay (ELISA)

Collagen I secretion from HK2 cells was quantified using an anti-procollagen I α1 ELISA (ab210966) according to manufacturer’s instructions. HK2 cells were incubated with TGFβ1 (10 ng/mL) ± Peptide 5 (25 µM) for 48 h. HK2 cell supernatant (50 µL) was added to each well of the pre-coated anti-procollagen I α1 96 well plate with antibody cocktail (50 µL) for 1 h at RT. Subsequently, each well was washed 3 times with 350 µL wash buffer before the addition of 3,3′.5,5′-Tetramethylbenzidine (TMB) development solution (100 µL) for 10 min. After this time, 100 µL stop solution was added prior to measuring the optical density (OD) at 450 nm. Final values were interpolated on a standard curve.

### 4.7. Carboxyfluorescein Dye Uptake Assay

HK2 cells were cultured on fluorodishes and incubated with TGFβ1 (10 ng/mL) ± anti-integrin α2β1 neutralising antibody (2.5 µg/mL) or Peptide 5 (25 µM) for 48 h. Cells were exposed to Ca^2+^-free (supplemented with EGTA (1 mM)) balanced salt solution (BSS) + carboxyfluorescein (200 µM) for 10 min to permit hemichannel-mediated dye uptake. This was followed by a 5-min incubation period with Ca^2+^-containing BSS + carboxyfluorescein (200 µM) to shut hemichannels and trap dye uptake. 3 × 10 mL washes with Ca^2+^-containing BSS removed residual carboxyfluorescein dye. A Cool Snap HQ CCD camera (Roper Scientific, Gottingen, Germany) along with Metamorph software (v7.75, Universal imaging Corp., Marlow, Bucks, UK) was used for image acquisition. A region of interest (ROI) was manually drawn around individual cells (~15 cells/image) to measure the mean integrated density using Fiji software (v1.0, imageJ, LOCI, Wisconsin, USA) as described in detail previously [86].

### 4.8. ATP Biosensing

ATP biosensors (Sarissa Biomedical, Coventry, UK) were used in simultaneous dual recording ampomeric mode as described previously [8]. A null sensor accounted for non-specific electro-active artefacts and was subtracted from the ATP trace. Glycerol (2 mM) was included in all solutions. HK2 cells were cultured on either uncoated or collagen I coated glass coverslips and incubated with TGFβ1 (10 ng/mL) for 48 h. Cells were transferred to a central chamber with a Ca^2+^-containing BSS + glycerol (2 mM) perfusion system at a rate of 6 mL/min (37 °C) and left for 10 min to acclimatise. This was switched to Ca^2+^-free BSS + glycerol (2mM) to open hemichannels, prior to closing with Ca^2+^-containing BSS + glycerol (2mM). Data was calibrated using 10 µM ATP. Recordings were acquired at 4Hz with a Micro CED (Mark2) interface using Spike software (Cambridge Electronic Design, UK) as described in detail previously [87,88].

### 4.9. Statistical Analysis

All data are presented as the mean ± SEM. Statistical analysis was performed using a one-way ANOVA or paired T-test where necessary with a Tukey’s multiple comparison post-test. A *p*-value < 0.05 represents significance, whilst ‘*n*’ denotes the sample number.

## Data Availability

The data presented in this study are available in either this article or in the Supplementary Material.

## Supplementary Materials

The following are available online at https://www.mdpi.com/article/10.3390/ijms22073644/s1.
