# Supplementary material for: Collagen I Modifies Connexin-43 Hemichannel Activity via Integrin α2β1 Binding in TGFβ1-Evoked Renal Tubular Epithelial Cells
Source: Int J Mol Sci. 2021 Mar 31;22(7):3644. doi: 10.3390/ijms22073644 (PMC8038016; doi:10.3390/ijms22073644)
Supplement: Supplementary file 1 [file ijms-22-03644-s001.pdf]

Supplementary material

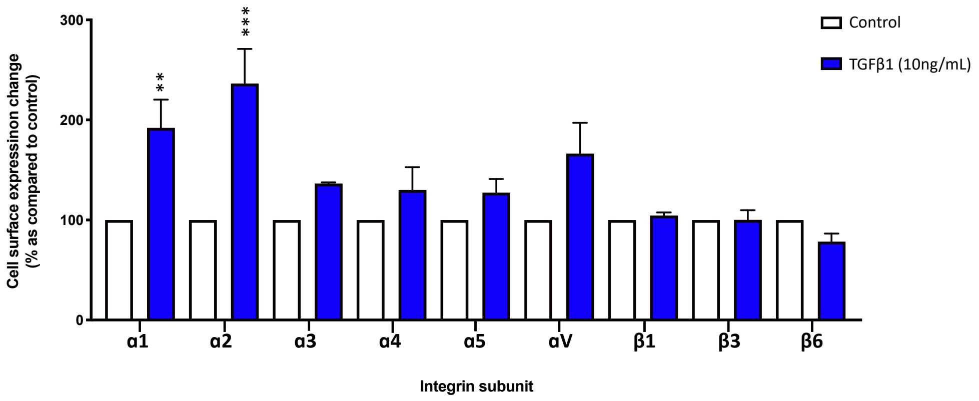

Figure S1. TGFβ1 increases cell surface integrin α1 and α2 expression.
HK-2 cells were supplemented in low (5mmol/L) glucose ± TGF-β1 (10ng/mL) for 48 hours and seeded on a Chemicon precoated integrin-mediated cell adhesion array plate. Changes in cell surface integrin subunit expression were assessed. Cells treated with TGF-β1 (10ng/mL) exhibited a significant increase in integrin α1 and α2 expression. Optical density results were as compared to control, from three separate experiments. BSA was used as a negative control. Key significance shown: **P<0.01, ***P<0.001.

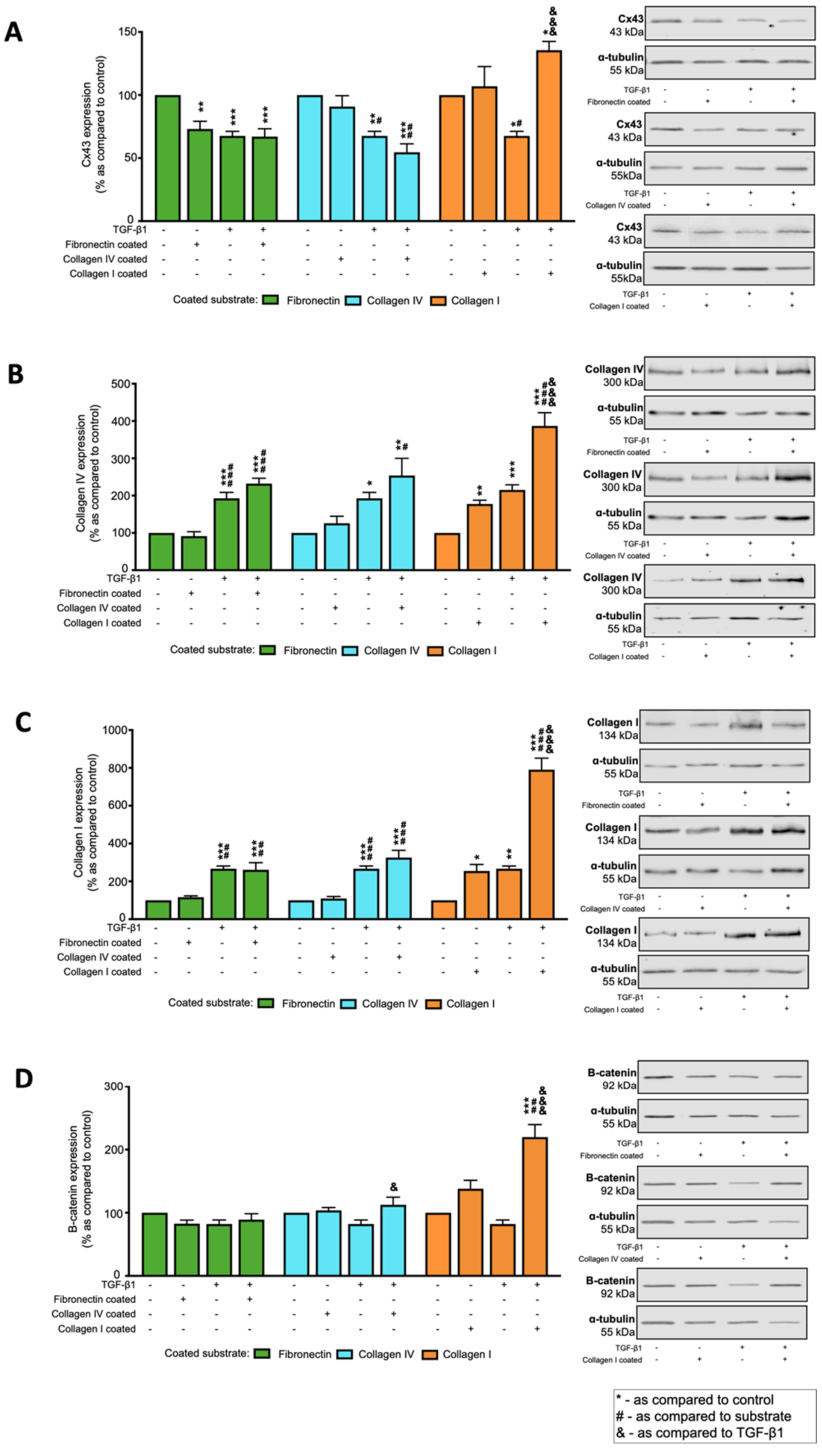

Figure S2. The effect of Fibronectin, Collagen IV and Collagen I +/- TGF-b1 (10ng/mL) on expression of markers of tubular injury . HK2 cells were cultured on uncoated, fibronectin, collagen IV or collagen I coated plastic in low (5mmol/L) glucose ± TGFβ1 for 48 hours. Whole cell expression of Cx43 (A), collagen IV (B), collagen I (C) and β-catenin (D) were confirmed *via* immunoblotting. Uncoated plastic served as a control. Representative blots for each protein are shown, with expression normalised against α-tubulin as a loading control. Results were from four or more separate experiments; with key significances shown: *P<0.05, **P<0.01, ***P<0.001 vs control; #P<0.05, ##P<0.01, ###P<0.001 vs substrate; &P<0.05, &&&P<0.001 vs TGFβ1.

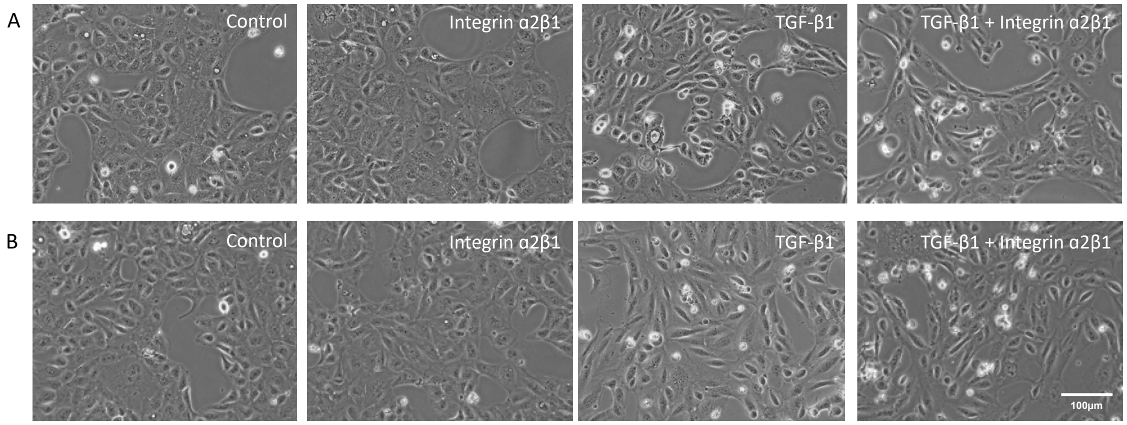

Figure S3. Inhibiting integrin α2β1 has no effect on cell morphology. HK2 cells were cultured on collagen I (50µg/mL) in low glucose (5mmol/L) ± TGFβ1 (10ng/mL) ± anti-integrin α2β1 neutralising antibody (2.5µg/mL) for 48 hours. Phase contrast microscopy assessed changes in cell morphology in cells cultured on uncoated plastic (A) and collagen I coated plastic (B) (magnification x20). Integrin α2β1 inhibition did not alter classic cobblestone epithelial control cell morphology in cells cultured on uncoated or collagen I coated plastic. TGFβ1 evoked elongation was also not affected by anti-integrin α2β1 neutralising antibody.
